# Cannabidiol Does Not Cause Significant Changes to Working Memory Performance in the N-Back Task

**DOI:** 10.3390/ph14111165

**Published:** 2021-11-16

**Authors:** Éamon Jones, Styliani Vlachou

**Affiliations:** Neuropsychopharmacology Division, Behavioural Neuroscience Laboratory, School of Psychology, Faculty of Science and Health, Dublin City University, Glasnevin, Dublin 9, D09 Y074 Dublin, Ireland; eamon.jones5@mail.dcu.ie

**Keywords:** cannabidiol, working memory, cannabinoid, cognition, N-back task

## Abstract

Cannabis use can be traced back to several centuries before the Common Era, when it was used for industrial, medicinal and recreational purposes. More recently, over 100 different cannabinoid compounds have been identified, one of which is cannabidiol (CBD), a compound widely used for anti-inflammatory and anxiolytic treatment. The literature surrounding the cognitive effects of CBD is limited, with most studies focusing on the effects of other cannabinoids on cognition. To expand this literature, this study investigated whether CBD causes significant differences to working memory (WM) functioning, as measured by the N-back task. It was hypothesised that CBD does not cause statistically significant differences to WM. In all, 54 participants, 33 females and 21 males, were recruited, with a mean age of 32.63 years. Of these 54 participants, 26 reported using CBD and no other cannabinoids, while 28 reported not using any cannabinoid. The participants were instructed to answer a short online survey to gather basic demographic data and to complete an online N-back task to measure WM. For the computerised N-back task, the participants completed a practice and three test blocks, where they were instructed to respond to whether a series of letter stimuli were presented one trial back (1-back), two trials back (2-back) or three trials back (3-back). Multivariate analysis of covariance yielded no statistically significant difference on either response time or response accuracy data between groups after controlling for how long the participants use CBD and for what reason they use CBD. These results support our hypothesis that CBD does not cause significant changes to WM functioning. Further research is greatly needed to investigate the long-term effects of CBD use on WM and on general cognitive functioning.

## 1. Introduction

*Cannabis sativa* is the most widely used variety of hemp, with some people ingesting cannabis to experience feelings of relaxation and to induce a sense of calm, while others ingesting the drug to reduce pain and anxiety. The first uses of cannabis can be traced back to China several centuries before the Common Era, where it was used to make rope and paper [1]. 

Cannabis remains one of the most widely used drugs in the world, with 15.7% of 15–24-year-olds using cannabis or cannabis products in the past year globally. This number falls to 10.6% in those aged between 25 and 34 years old and to less than 6% in those older [2]. The most common method of ingesting the drug is by smoking the cannabis flower in a cigarette or water pipe [3]. 

Cannabinoid compounds can be found in varying levels throughout the plant; however, the plant grows a flower coated in a thick waxy resin where the levels of over 100 cannabinoids are the highest [4]. Among other cannabinoid compounds, the flower is composed of two main compounds: the psychoactive Δ^9^-tetrahydrocannabinol (Δ^9^-THC), discovered by Gaoni and Mechoulam in 1964 [5], and the non-psychoactive cannabidiol (CBD), which was isolated in 1940 [6] and successfully elucidated by Mechoulam and Shvo in 1963 [7]. 

The human endocannabinoid system is highly complex and consists of at least two types of class A G-protein-coupled receptors (GPCRs): the cannabinoid one receptor (CB_1_) and the cannabinoid 2 receptor (CB_2_) [8]. The body produces endogenous ligands to these receptors, with the first being identified in 1992: N-arachidonoylethanolamide, named anandamide for short after the Sanskrit word ananda, meaning bliss [9]. Some years later, in 1995, a second endogenous cannabinoid was identified: 2-arachidonoyl-glycerol (2-AG) [10,11,12,13,14,15,16]. These endogenous cannabinoid ligands are produced immediately before excitatory release from fatty compounds in the cell membrane of neuronal cells and are released mostly from the cell body and dendrites, where they exert their effects on presynaptic neurons [14]. The cannabinoid receptors can be found mainly concentrated in the spinal cord and the brain, primarily in the basal ganglia and cerebellum, and in corticolimbic areas, such as the cingulate cortex, amygdala and hippocampus [17,18]. This is consistent with the characteristic psychoactive effects of Δ^9^-THC as these brain areas are implicated in movement control, decision making and emotional and cognitive processes, all of which are impaired by Δ^9^-THC. Outside of the central nervous system (CNS), cannabinoid receptors can be found in abundance in the gastrointestinal tract, in adipose and connective tissues and in the liver [17]. Δ^9^-THC has a high affinity for the cannabinoid receptors, whereas CBD has little affinity for either CB_1_ or CB_2_ receptors; CBD acts as an inverse agonist of CB_1_ [8], while it also has an affinity for 5-HT_1A_ and 5-HT_2A_ receptors and binds to peroxisome-proliferator-activated receptor gamma (PPAR-γ), which plays a role in neuroprotection and anti-inflammation [18]. Additionally, CBD promotes peroxisome-proliferator-activated receptor alpha (PPAR-α) activity by inhibiting fatty acid amide hydrolase (FAAH) [19]. As mentioned, CBD has low binding affinity for either CB_1_ or CB_2_, which may account for its non-psychotropic characteristic. CBD is considered a multi-target compound. At low micromolar to sub-micromolar concentrations, CBD inhibits the orphan G-protein-coupled receptor GPR55, the equilibrative nucleoside transporter (ENT) and the transient receptor potential of melastatin type 8 (TRPM8) channel [10]. Conversely, CBD amplifies the activity of the 5-HT_1A_ receptor, the transient receptor potential of ankyrin type 1 (TRPA1) channel and the a3 and a1 GlyRs, and it has a bidirectional effect on intracellular calcium [20]. At larger micromolar concentrations, CBD acts through receptor-independent channels and by binding with non-cannabinoid receptors. Extensive studies show that a large portion of the ingested amount of CBD is excreted intact or as one of its metabolites through a process known as glucuronidation, a chemical reaction that occurs during phase 1 metabolism [21]. Because of this extensive phase 1 metabolism, the bioavailability of CBD seems to be relatively low [22,23,24,25], with the elimination half-life of CBD ranging between 2 and 5 days [26,27], bringing how effective CBD would be as a long-term treatment into question.

Compared to the most heavily studied intoxicating compound Δ^9^-THC, CBD was initially believed to be an inactive compound, i.e., having no effects on the body whatsoever. However, several years after the compound was discovered, it was found that CBD does indeed have different effects on the body [28,29]. CBD has since been used to treat a variety of conditions; for example, Chagas et al. [30] reported that administering either 75 mg or 300 mg of CBD for a period as short as 6 weeks significantly reduces the amount of rapid eye movement sleep disturbances in individuals with Parkinson’s disease. More frequently, CBD has been used as an alternative treatment to reduce the symptoms of anxiety and compulsive disorders [31]. The logic behind this stands true as CBD binds primarily to 5-HT_1A_ and additionally to 5-HT_2A_ receptors, which are also the binding sites of many anxiolytic drugs, such as buspirone [31,32]. This finding supports the results of previous studies investigating the anxiolytic effects of CBD when taken in combination with Δ^9^-THC [33,34,35].

There is some evidence to suggest that cannabinoids are beneficial to the functioning of working memory (WM). It is suggested that when used in combination, CBD protects memory functioning against the harmful effects of large doses of or the chronic use of Δ^9^-THC [34,35,36]. It is suggested that these neuroprotective effects of CBD can be explained due to its anti-inflammatory and anti-oxidative properties [36].

Currently, there is limited research focusing on the cognitive effects of CBD alone, with most of the research in the field of cannabinoids and cognitive functioning being concerned with either the effects of Δ^9^-THC alone or the effects of the combination of Δ^9^-THC and CBD [6,34,37,38,39,40]. Hindocha et al. [41] conducted a study to investigate whether the non-intoxicating cannabinoid CBD shows pro-cognitive effects in individuals experiencing nicotine withdrawal. Thirty non-treatment-seeking smokers attended two laboratory sessions after an overnight nicotine abstinence period, where they were orally administered either 800 mg of CBD or a placebo in a double-blind randomised manner. Cognitive functioning was assessed by administering a series of memory and impulsivity tests, such as the go/no-go task, the delay discounting task and the prose recall task. The authors reported that the use of CBD had no significant difference to the cognitive functioning of the nicotine withdrawal group compared to the control group, as measured by the delay discounting task and the prose recall task. Contrary to what they had hypothesised, it was found that those who were administered CBD displayed increased commission errors on the go/no-go task, suggesting that CBD had a negative effect on the participants’ cognitive functioning during nicotine abstinence. These results do not indicate definitively whether CBD causes positive or negative changes in WM functioning. It is important to consider in this study that participants were administered only a single dose of CBD after a short nicotine abstinence period. 

Similar results were found by Bhattacharyya et al. [33], who investigated the effects of 10 mg of Δ^9^-THC, 600 mg of CBD or a placebo on brain activity, as measured by functional magnetic resonance imaging (fMRI) in healthy participants performing a verbal memory task. The authors reported that neither Δ^9^-THC nor CBD had an effect on the cognitive functioning of these participants. Interestingly, the authors reported that CBD augmented striatal, anterior cingulate, medial and lateral prefrontal cortical activation during the verbal memory task and also increased activation in the parahippocampal gyrus, left insula and caudate nucleus during a response inhibition task. 

Surrounding the investigation of the effects of CBD on WM, the results of these studies reflect a common trend in the literature in that they are conflicting and do not point definitively to whether CBD can indeed cause WM deficits or show pro-cognitive effects and improve WM. To help expand the knowledge on the potential impact of CBD in WM, this study intends to investigate whether the use of CBD causes changes to WM in a non-clinical population sample, as measured by the N-back test. 

For this study, the participants were presented with an N-back task, originally described by Kirchner in 1958 [42], following a short questionnaire. In our study, participants were presented with a computerised version of the N-back task, and they were instructed to respond to whether the stimulus currently presented was salient or conflicting with the stimuli presented a specified number of tasks ago. Based on existing literature, it was hypothesised that participants who use CBD sublingual drops or tablets will not perform statistically significantly different on either reaction time (RT) data or accuracy measures of the N-back test than those who do not use CBD.

## 2. Results

### 2.1. Demographics

A total of 68 individuals participated in this study, of which only 9 CBD users partially completed the experiment as did 5 non-CBD users. Their data were excluded from statistical analysis. A total of 54 individuals fully completed this study, 33 females (61.1%) and 21 males (38.9%), with a mean age of 32.6 years (SD = 14.32 years). Twenty-eight participants (51.85%) reported that they did not use CBD or any other cannabinoid and were deemed to be members of the control group. Of the 26 participants (48.15%) who reported that they did use CBD, 13 reported using CBD sublingual drops only and 13 reported using CBD tablets. No participants reported using more than one CBD product concurrently. Of these 26 individuals, most reported using CBD to aid sleep. See Table 1 for a breakdown of the reported uses of CBD by the recruited participants. The mean strength of CBD used among participants was 541.35 mg (SD = 575.8 mg). 

### 2.2. WM Performance 

A one-way multivariate analysis of covariance (MANCOVA) was conducted with the RTs for the 1-back, 2-back and 3-back trials and response accuracy on the 1-back, 2-back and 3-back trials as dependent variables; whether they used CBD or not as independent variables; and the reason for CBD use, duration of CBD use (see Table 2 for a breakdown of how long participants reported using CBD) and the strength of CBD used as covariates in order to control for dose effects and expected cognitive differences in known groups that used CBD.

None of the data tested violated the assumptions of normality, linearity, univariate and multivariate outliers, homogeneity of variance–covariance matrices, multicollinearity, independence of the covariates from the experimental treatments and reliability of the measurement of the covariates, so the data were deemed suitable for MANCOVA. The alpha level was set to *p* < 0.05. No statistically significant difference between group means on the dependent variables after controlling for covariates was found: F(12, 86) = 0.716, *p* = 0.732; Wilk’s lambda = 0.826; and ηp^2^ = 0.091. Since these analyses yielded no statistically significant differences of any group on the dependent variables after controlling for covariates, no further post hoc tests of between-subject effects were deemed necessary. 

**Figure 1:**
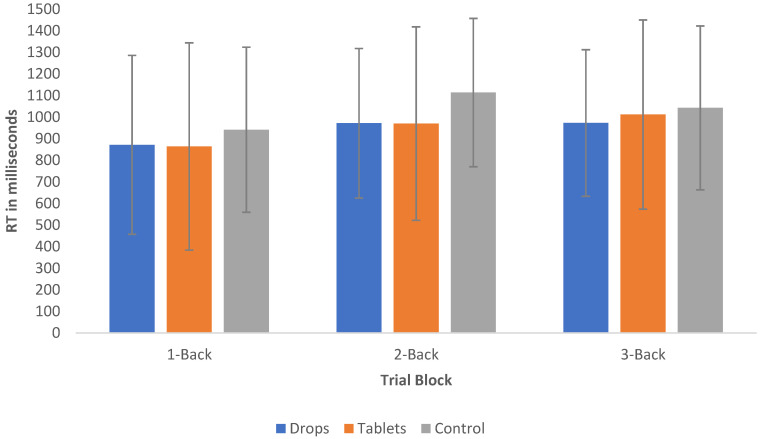
Mean RT and standard deviation of the 1-back, 2-back and 3-back trials of CBD sublingual drop users, CBD tablet users and the control group.

**Figure 2:**
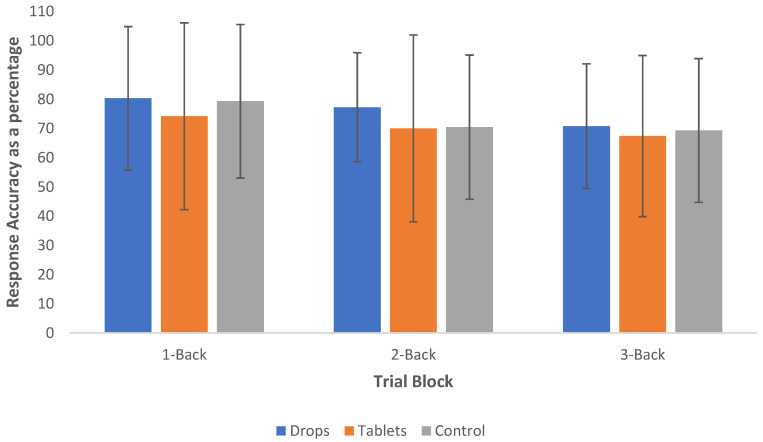
Mean response accuracy and standard deviation of the 1-back, 2-back and 3-back trials of CBD sublingual drop users, CBD tablet users and the control group.

Interestingly, the mean RT of controls (M = 1032.34, SD = 69.61) was between 84 and 94 ms longer than that of who use CBD tablets (M = 948.25, SD = 126.32) and sublingual drops (938.33, SD = 101.66), respectively, but this failed to meet statistical significance; see Figure 1 for a visual representation of the mean RTs for both groups, and see Figure 2 for a visual representation of the accuracy of responses of both groups. With regard to the accuracy data, the mean of correct responses in the control group was 73% (SD = 5.96%), 70.51% (SD = 8.46%) in the CBD tablet group and 76.10% (SD = 5.96%) in the sublingual drop group.

## 3. Discussion

It was hypothesised that CBD does not cause significant differences in WM functioning. The data collected were robust, with the RTs and number of errors observed to increase as the cognitive load increased. As was hypothesised, analysis of the results yielded no statistically significant difference between the RT or accuracy of CBD users in the N-Back Task compared with controls. Similarly, no statistically significant result was found regarding the strength of CBD used, suggesting that there is not a dose-dependent relationship with N-back performance. Interestingly, it was noted that the mean RT of controls was up to 94 ms longer than that of CBD users. However, those in the control group responded less accurately than those in the CBD groups. Neither of these results reached statistical significance. 

The findings of this study are both salient and conflicting with the small amount of the available literature. Consistent with the results found here, Hindocha et al. [41] reported that CBD does not cause significant changes in memory as measured by the delay discounting, prose recall and N-back tests, but CBD caused significant changes on the go/no go task. This finding conflicts with this study in that no cognitive deficits were observed. Salient to our findings, Bhattacharyya et al. [33] reported that CBD does not cause negative changes to the participant’s memory functioning and reported increased cortical activation in CBD users. However, this increased activation may have occurred as an anti-oxidative response to Δ^9^-THC. 

### Strengths and Limitations

As with almost all scientific research, this study had several limitations. Namely, due to the current COVID-19 emergency and government restrictions on social interaction, it was impossible to conduct the N-back task in a controlled experimental environment. It would have been preferable to conduct this study in a quiet examination room with nothing but the test computer with the survey and task already loaded. This would prevent the possibility of the N-back task website loading slowly and prevent a computer glitch. The participants were instructed in a plain language statement to complete the task in a quiet room with a reliable internet connection, but this could not be verified. This may indirectly have been an advantage, as experimental settings have been known to increase levels of anxiety, worry and general unease, which could cause a significant interference in test performance [43], whereas completing the task in a familiar setting is less likely to evoke these emotions, thus creating a calmer environment and more accurate results.

Another limitation was the lack of verifiability of the quality or strength of products used. In an ideal situation, participants would be instructed to use varying strengths of one particular brand or company, which has been laboratory-tested with verifiable quality and strength. However, the consumer market has experienced a significant influx of CBD products, some of which have unlicensed and unverified claims as well as questionable quality and potency. Continuing with this, it cannot be ensured that the levels of Δ^9^-THC are below the legal limit of 0.3% for cannabinoid products in the Republic of Ireland. If participants have consumed products with Δ^9^-THC at a level higher than what is disclosed on the labelling and packaging, the results may be skewed and inaccurate.

Similarly, having more than one route of administration could be considered a limitation. In this study, the participants who used CBD were split by either those who use CBD sublingual drops or those who use CBD tablets. This was done as CBD tablets must undergo first-pass metabolism before any benefit is observed. As drops bypass first-pass metabolism, it was thought that more CBD could be absorbed; however, further studies are needed to clarify this.

A further limitation of this study was the fact that the information regarding other medications that were currently used by the participants was not collected. Because of this, it could not be guaranteed that some participants were not taking medication that may have influenced their WM performance. This was done, in part, due to the large volume of drugs that affect memory and wider cognitive functioning. 

The reliability of the N-back task as a measure of WM must also be considered as a possible limitation. Similar to the literature surrounding the relationship between CBD and WM, the literature surrounding the reliability of the N-back task is unclear and confusing, with authors such as Jaeggi et al. [44] and Kane et al. [45] suggesting that the N-back task should not be used in experimental settings, as it does not hold convergent validity with other WM paradigms but holds face validity as a WM task. Miller et al. [46] suggested that the N-back task is not a pure measure of WM but may in fact be useful in research to measure subtle differences between groups. There is an equal amount of data supporting the use of the N-back task as a measure of WM in experimental settings. Kearney-Ramos et al. [47] reported that the letter variant of the N-back task, as was implemented in this study, holds more reliability as a measure of WM when compared to the number variant of the N-back or the dual N-back task or other well-established measures, such as the Halstead–Reitan finger-tapping test or the Boston Naming Test. Similarly, Jacola et al. [48] reported that the N-back task has good validity in measuring WM during functional magnetic resonance imaging and provides meaningful data.

Despite these limitations, this study had some clear strengths. The online software used, Psytoolkit, provides accurate RT and accuracy data and is well supported by the literature [44,49,50,51]. Psytoolkit is especially useful as it does not require participants to download any applications or files, as would be the case with applications such as Pavlovia. All that is required of participants is to simply click a link to a webpage for the task to load. Another advantage of this study was that the participants were recruited from various locations. Furthermore, the data collected were from a wide range of participants aged between 19 and 61 years who either do not use CBD or use CBD for varying reasons. Given that the literature surrounding the cognitive effects of CBD is so sparse, a significant advantage of this study was that it expanded the literature in a previously poorly studied area and supported the notion that this non-psychotropic compound does not cause harm or significant improvements to working memory functioning. 

Future studies in this area may consider focusing their attention on examining the wider effects of CBD on cognitive functioning as the literature in this topic is also severely lacking. Ideally, a complete neuropsychological battery, such as the Halstead–Reitan [49] or the Luria–Nebraska Neuropsychological Battery [50], would be administered at baseline to assess for differences between groups and then administered again after a prolonged period of CBD use to assess the differences between groups, controlling for normal factors such as age and disease (if administered to a clinical population). As mentioned previously, CBD has been thought to both alleviate and prevent the deficits associated with Δ^9^-THC use [33]. It would be both interesting and beneficial to expand the literature in this field by examining the effects of using CBD to ameliorate the effects of long-term cannabis or Δ^9^-THC use in non-clinical samples. This would provide further insight into the therapeutic potential of the compound, as well as providing insight into how the non-intoxicating cannabinoid CBD exhibits its benefits. 

This research is valuable as it provides insight into the effects of CBD on WM and general cognitive functioning. As there is no literature currently available studying the effects of CBD in a healthy population sample, or in those without a neurological condition; at the time of writing, one study was identified studying the effects of CBD on the WM of patients with treatment-resistant epilepsy. Similar to the results of our study, these authors reported that CBD does not cause statistically significant changes to WM functioning [51]), this information is important in informing patients and consumers on whether this compound will affect their WM. In addition, this study contributes greatly to the literature regarding a previously sparsely studied aspect of CBD by providing evidence to suggest that CBD does not cause changes to WM. 

## 4. Materials and Methods

An *a priori* power analysis was conducted using G*Power [52,53], which revealed that a sample size of 52 would be sufficient to detect a significant interaction effect with a power of 0.80 and an alpha of 0.05, as recommended by Cohen [54]. 

In all, 54 participants, 21 males and 33 females, with a mean age of 32.63 years (SD = 14.32), who had normal or corrected-to-normal vision, were recruited to participate in this study. There were two groups in this study: group 1 was composed of those who use CBD and no other cannabinoid, and group 2, i.e., the control group, was composed of individuals who do not consume CBD or any other cannabinoid. 

Recruitment flyers that contained relevant information and a link to the website where the participants could find the plain language statement, the researcher’s contact information and the informed consent sheet were displayed in pharmacies and health food shops in County Wexford, Ireland. An online version of the recruitment flyers was also posted on online CBD forums, such as r/CBD, r/Crainn, r/CBDinfo, r/CBDoil and r/CBD_UK on the social media platform Reddit, with the forum administrators’ permission. The information was also posted on a similar forum page on Facebook, the CBD Support Group Ireland, once permission was granted by the forum administrators. Due to the coronavirus disease 2019 (COVID-19) emergency, in line with public health guidelines at the time of recruitment, in-person measures of recruitment were not possible.

### 4.1. Inclusion and Exclusion Criteria

Prospective participants were deemed eligible for inclusion if they met the following criteria: (1) were aged 18 years or older, (2) had normal or corrected-to-normal vision and (3) provided informed consent after reading the plain language statement and informed consent sheet. Prospective participants were excluded from this study if they (1) were not aged 18 years or older, (2) did not have normal or corrected-to-normal vision, (3) were colour-blind, (4) had dyslexia or other disorders that may affect how they process visual stimuli and (5) smoked cannabis or used products containing ∆^9^-THC or any other cannabinoid compounds. 

### 4.2. Procedure

Once participants followed the link on the recruitment flyers or online recruitment posts, they were presented with a plain language statement that detailed why the study was being conducted, what was required of them and the contact details of the researchers. If the participant was happy to proceed, they clicked forward to the informed consent sheet. This informed consent sheet again briefly described what the study entailed, what was required of them and the contact information of the researchers. The sheet asked the participants to confirm that they understood that their participation was voluntary, they were aged at least 18 years old, they were aware they could terminate their participation at any time up until completion of the N-back task, they understood that their data were anonymous and they confirmed that they did not have any conditions affecting how they process visual stimuli. 

Next, the participants were presented with a short questionnaire that asked basic demographic questions regarding age and gender. The questionnaire also asked whether they use CBD (if so, whether they use sublingual drops or tablets), why they use CBD, how long they have used CBD for and the potency of the CBD product they use. At the end of this questionnaire, the participants were provided with a link to the online N-back task. 

The N-back test was performed on the participants’ own computers in a quiet environment, as instructed, using the online psychology software Psytoolkit [55,56]. The task was coded by one researcher on the Psytoolkit platform using the computer programming language Python. The participants were asked to identify whether they saw a stimulus immediately previously (1-back), two trials previously (2-back) or three trials previously (3-back). 

For the current N-back task, only visual stimuli, as described previously by Buschkuehl et al., Schmid et al. and Jaeggi et al. [57,58,59], were used for this study. The participants were presented with one practice block, which consisted of 15 trials, followed by three test blocks (1-back, 2-back and 3-back) with 25 trials per block. Stimuli consisted of 15 letters: A, B, C, D, E, H, I, K, L, M, O, P, R, S and T. These 15 letters were used in favour of the first 15 letters of the alphabet in sequence as they do not look remarkably similar to each other, preventing confusion and participants responding by mistake to a letter that looks similar to the target stimulus. Each trial was presented for 2000 ms or until the participant responded, which would prompt the presentation of the next trial. The participants responded by pressing the “m” key if they had seen the stimulus n-trials back or the “n” key if they had not seen the stimulus n-trials back. The participants received direct feedback as to whether they responded correctly, incorrectly or not at all to each trial by small bars surrounding the stimulus turning either green for a correct answer and red for an incorrect answer or for answering too slowly. Before each block, an instructions page was presented describing how to respond to the stimuli, which stimuli they are to respond to (e.g., 1-back, 2-back or 3-back), and a visual example of a trial. 

Once participants finished the N-back task, the screen would change to a debriefing sheet that would once again detail the purpose and aims of the study, provide the contact information of the research supervisor and the Chairperson of the Psychology Ethics Committee (PEC) of Dublin City University and the contact information of free-to-contact emergency mental health services should the participants experience negative emotions as a result of taking part in this research. 

The survey portion of the experiment including the plain language statement, informed consent form and demographic questions took roughly 3 min to complete, while the N-back task portion of the study took roughly 6 min for the participants to complete.

Raw anonymised response time and accuracy data were extracted from the online Psytoolkit server, and raw anonymised survey responses were extracted from the online Qualtrics server. The data were cleaned and assigned values in Microsoft Excel before being extracted into a new SPSS file. IBM SPSS 27th version for Mac was used for subsequent data analysis. The data underwent one-way multivariate analysis of covariance (MANCOVA) for the purpose of investigating the primary outcome of whether using CBD causes a statistically significant difference to WM functioning of controls. 

## 5. Conclusions

This study was conducted in order to evaluate whether CBD causes a statistically significant change in WM functioning. No statistically significant difference in WM functioning was found in those who use CBD compared to those who do not use CBD. Further research is greatly needed to assess the effects of CBD on wider cognitive functioning.

## Figures and Tables

**Table 1:** The reasons for CBD use reported by the participants.

Reason for Use	Frequency	Percentage of CBD User Group	Percentage of Total Sample
Does not use CBD	28	0%	51.85%
Migraine relief	2	7.70%	3.70%
To improve mood	7	26.92%	12.96%
For pain relief	7	26.92%	12.96%
To aid sleep	10	38.46%	18.51%
Total	54	100%	100%

**Table 2:** The length of CBD use reported by the participants.

Length of Use	Frequency	Percent of CBD User Group	Percentage of Total Sample
Does not use CBD	28	0%	51.85%
Less than 1 year	7	26.92%	12.96%
For 1 year	10	38.46%	18.52%
For 2 years	7	26.92%	12.96%
For 3 years	2	7.70%	3.70%
Total	54	100%	100%

## Data Availability

Data is contained within the article.
